# MEASURING DISTRESS IN DEMENTIA: A PILOT STUDY ON PERCEIVED MEMORY, DYADIC RELATIONSHIPS, AND SELF-EFFICACY

**DOI:** 10.1093/geroni/igad104.3465

**Published:** 2023-12-21

**Authors:** Amanda MacNeil, Katherine Judge

**Affiliations:** Cleveland State University, Cleveland, Ohio, United States; Cleveland State University, Cleveland, Ohio, United States

## Abstract

Prior literature has suggested that some individuals with dementia are capable of self-reporting details of their lived experience. With this, researchers have been increasingly interested in understanding what it is like to live with dementia from the individual’s perspective. To capture a more holistic image of the illness experience of individuals with dementia, it is important to consider how they perceive and cope with various aspects of their illness. While some work has suggested that the perceptions that individuals with dementia have about their illness are important to things such as well-being outcomes, no prior work has addressed the distress that results from these perceptions. This pilot study (N = 26) examined perceptions of difficulties and feelings of distress related to three different areas of the illness: one’s memory, the relationship with their caregiver, and self-efficacy. Results suggest that perceived memory distress (α = 0.71), dyadic relationship strain distress (α = 0.80), and self-efficacy distress (α = 0.82) are reliable measures when used in a sample of individuals with mild to moderate dementia (MMSE M = 20.5, SD = 5.20). Interestingly, some of these measures may even be related such as perceived memory distress and self-efficacy distress (r (24) = .50, p = .01). Including distress felt because of perceiving difficulties in various areas of one’s illness adds a layer of understanding to the illness experience. This can aid in addressing well-being outcomes and serve as intervention targets in future work.

